# Cryptic animal species are homogeneously distributed among taxa and biogeographical regions

**DOI:** 10.1186/1471-2148-7-121

**Published:** 2007-07-19

**Authors:** Markus Pfenninger, Klaus Schwenk

**Affiliations:** 1 Abteilung Ökologie & Evolution, J.W. Goethe-Universität, Biologie Campus Siesmayerstraße, 60054 Frankfurt am Main, Germany

## Abstract

**Background:**

Cryptic species are two or more distinct but morphologically similar species that were classified as a single species. During the past two decades we observed an exponential growth of publications on cryptic species. Recently published reviews have demonstrated cryptic species have profound consequences on many biological disciplines. It has been proposed that their distribution is non-random across taxa and biomes.

**Results:**

We analysed a literature database for the taxonomic and biogeographical distribution of cryptic animal species reports. Results from regression analysis indicate that cryptic species are almost evenly distributed among major metazoan taxa and biogeographical regions when corrected for species richness and study intensity.

**Conclusion:**

This indicates that morphological stasis represents an evolutionary constant and that cryptic metazoan diversity does predictably affect estimates of earth's animal diversity. Our findings have direct theoretical and practical consequences for a number of prevailing biological questions with regard to global biodiversity estimates, conservation efforts and global taxonomic initiatives.

## Background

Cryptic species are two or more distinct species that were classified as a single species due to their morphological similarity. Facilitated through technical advances such as PCR and direct DNA sequencing, many phylogenetic, phylogeographic and population genetic studies in the last two decades discovered – frequently without *a priori *intention – genetically divergent but morphologically cryptic lineages. These discoveries continue to increase exponentially and raise a number of general questions, such as: How frequent are cryptic species? Are cryptic species evolutionary young? Is morphological stasis upon speciation more often found in environmental extremes, such as the tropics, the artic or the deep sea? A recent review proposed that the distribution of cryptic species is non-random across taxonomic groups and biomes, which might have substantial consequences for biodiversity assessments, macroecology, biogeography, conservation management and evolutionary theory [1]. Biodiversity estimates of certain taxonomic groups might be largely underestimated, ecological interactions remain hidden, conservation efforts may be inappropriate and cryptic pathogens, parasites and invasive species might represent unrecognised threads to human health. To tackle these issues, we need more information on the proportion of cryptic species in different phyla and different biomes. Here, we analysed the Zoological Record™ database (1978–2006) for the taxonomic and biogeographical distribution of cryptic metazoan species in relation to the number of described species.

## Results and discussion

**Figure 1:**
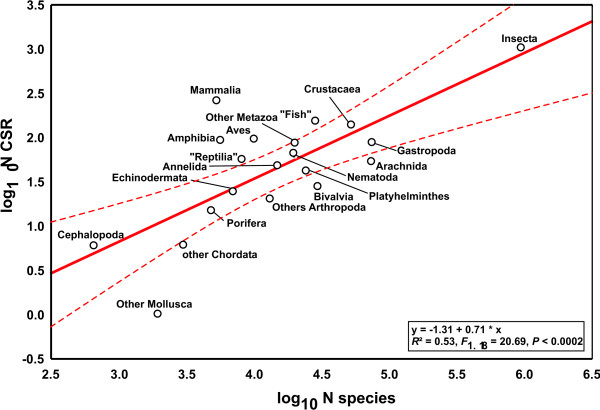
**The log_10 _of cryptic species reports (CSR) as a function of the log_10 _number of described species in the respective taxon**. Deviations from the regression line represent CSR taxon variation. Dashed lines represent 95% confidence intervals.

**Figure 2:**
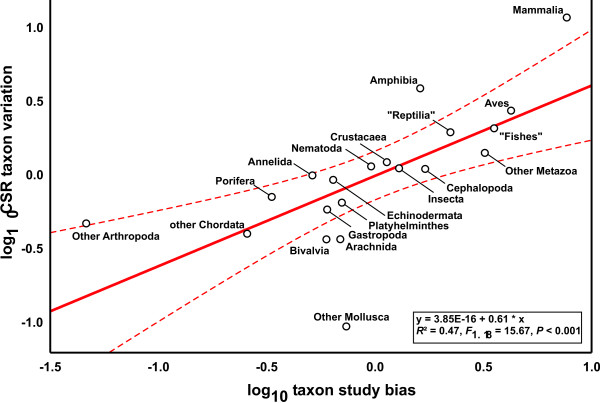
**Regression of CSR taxon variation on taxon study bias for 19 metazoan taxa**. Dashed lines represent 95% confidence intervals.

We found 2,207 cryptic species reports (CSR) under 771,931 studies that were suitable to detect cryptic species. Log_10 _number of CSR were correlated with the log_10 _of estimated number of described species in different metazoan taxonomic groups (Fig. 1, *R*^2 ^= 0.53, *F*_1,18 _= 20.69, *P *= 0.0002). Deviations from the regression line, termed CSR taxon variation, are presumably composed of differences in study intensity and taxonomic practice in the respective research community, true differences among taxonomic groups and random error. In order to assess the impact of differential research intensity, we regressed the log_10 _number of studies on the log_10 _number of described species in the respective taxonomic group; the residuals were used as a measure of taxonomic study bias (*R*^2 ^= 0.64, *F*_1,18 _= 8.63, *P *= 0.0088). This parameter explained part of the CSR taxon variation (Fig. 2, *R*^2 ^= 0.47, *F*_1,18 _= 15.67, *P *= 0.0009). Assuming that the numbers of CSR are proportional to the true number of cryptic species, their distribution is nearly homogeneous across the taxa analysed. Only six groups fell outside the 95% confidence intervals: Mammalia, Amphibia and combined smaller Arthropoda classes exhibited an excess of CSR, while Bivalvia, Arachnida and combined smaller Mollusca classes exhibited a deficit of CSR. At least for vertebrates, we suspect that these results might result from taxonomic inflation [2]. Overall, differential taxonomic practice in the various research communities seemingly exerted no major impact on the results.

**Figure 3:**
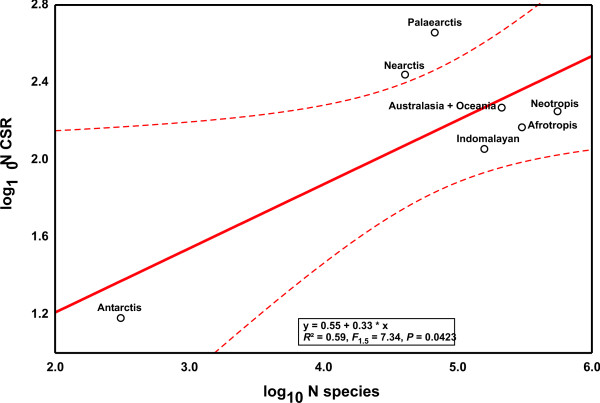
**The log_10 _of CSR as a function of the log_10 _number of described species in the respective region**. Deviations from the regression line represent CSR region variation. Dashed lines represent 95% confidence intervals.

**Figure 4:**
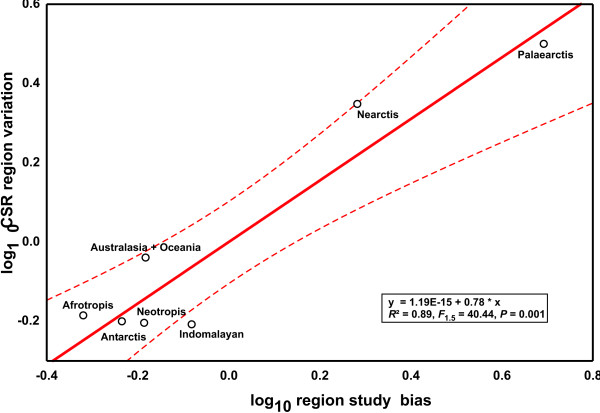
**Regression of CSR taxon variation on biogeographical region study bias**. Dashed lines represent 95% confidence intervals.

Applying the same procedure to the classic biogeographical regions revealed a marginally significant correlation between the number of CSR and the proportion of described species in the respective region (Fig. 3, *R*^2 ^= 0.59, *F*_1,5 _= 7.34, *P *= 0.0423). Study intensity of biogeographical regions was independent of estimated metazoan species richness (*R*^2 ^= 0.41, *F*_1,5 _= 3.41, *P *= 0.1242). However, regressing the residuals against each other, the same nearly neutral pattern emerged (Fig. 4, *R*^2 ^= 0.89, *F*_1,5 _= 40.44, *P *= 0.0014), which contradicts the view that tropical regions harbour relatively more cryptic species [1].

## Conclusion

Our results indicate that the proportion of cryptic species is almost evenly distributed among major metazoan taxa and biogeographical regions when corrected for species richness and study intensity. All users of taxonomic information must consequently be aware of the potential presence of cryptic diversity, regardless of taxonomic group or study area in focus. Further studies will show whether this relation holds for lower level systematic categories and other kingdoms.

Species are cryptic to human perception largely due to the lack of conspicuous differences in outward appearance. Given their homogeneous systematic and geographic distribution, it seems therefore that morphological stasis upon speciation represents an evolutionary constant, independent of phylogenetic relation or ecological circumstances.

Although the true proportion of cryptic species in nature is unknown, our results indicate that it seems to be similar across major metazoan taxa and biogeographical realms. Therefore, global barcoding initiatives [3], aiming at the exhaustive treatment of selected taxa like birds or fishes could also provide a first glimpse on the real extent of cryptic diversity in all metazoans.

The most important consequence of our unexpected finding is, however, that cryptic metazoan diversity can be treated as random error in biodiversity assessments [4]. There are probably not systematically more cryptic species among insects than in reptiles or in the tropics than in temperate regions. It stresses therefore that we should not preferentially target specific taxonomic groups or regions to detect cryptic species, but rather expect a predictable proportion of cryptic diversity in each metazoan group.

## Methods

The Zoological Record™ (Thomson Scientific) 1978–2006 was searched for entries containing the search term [cryptic speci* OR cryptic linea* OR cryptic tax* OR sibling spec*] which yielded 2207 entries. Even though the term sibling species has also the slightly different connotation of recent divergence [5], it is used synonymously to cryptic species in the great majority of cases. As cryptic species discovery is typically not an explicit aim in itself but a by-product of other research, we selected a reference data set of studies where the detection of cryptic taxa was possible. To this end, we scanned the retrieved cryptic species data set for typical keywords. These were sorted after approximate frequency and consecutively added to a query until more than 90% (1990 entries) of the initial data set were recovered. The discrepancy to 100% can be explained by the occasional absence of abstract and keywords as well as some articles being not in English. The final search string, finding 771,931 entries, was [phylogen* OR taxonom* OR systemat* OR morpholog* OR tax* OR discover* OR phylogeo* OR population genet* OR kary* OR genetic* struct* OR population struct* OR population different* OR speciat* OR identificat* OR diversi* OR descript* OR new spec* OR biodiv* OR spec* complex OR different* OR revision* OR molecular* OR distin* OR genet* var* OR radiat* OR species group OR species flock OR diagnostic OR status OR sympatr* OR survey]. The database records contain information about taxonomic affiliation and biogeographical origin, which allowed retrieving the number of studies performed on certain taxa and biogeographical regions. In general, our database searches were focussed on the larger phyla within the kingdom Metazoa. For the three most specious phyla (Arthropoda, Chordata, Mollusca), we analysed the major classes separately (Table 1). Information on the estimated number of currently described species for each taxon was obtained from the IUCN 2004 report [5] and various internet resources. The total number of metazoan species as well as their distribution across taxa closely matched numbers previously published [6]. No direct information on the species richness of biogeographical regions was available. However, the proportional species richness of various taxa occurring in the same biogeographical region shows substantial consistency [7]. We estimated therefore the proportion of species harboured in each region by averaging the proportion of mammals, amphibian and bird species as given in [5] as proxies for total faunal richness (Table 2). Data were log-transformed prior to standard regression analysis.

**Table 1:** Results of database search in Zoological Record™ (1978–2006) for several metazoan taxa. CSR = cryptic species reports.

**Taxon**	**N CSR**	**N articles**	**N species**
Arthropoda			
Insecta	996	219,754	950,000
Crustacaea	137	45,669	52,000
Arachnida	53	31,186	73,000
Other Arthropoda classes	20	885	13,000
Chordata			
Mammalia	267	92,592	5,416
Amphibia	92	20,243	5,743
"Reptilia"	58	33,130	8,163
Aves	94	69,899	9,917
"Fishes"	151	97,405	28,500
other Chordata classes	6	2,325	3,025
Mollusca			
Cephalopoda	6	7,791	768
Bivalvia	28	17,560	30,000
Gastropoda	86	27,794	75,000
Other Mollusca classes	1	5,407	1,950
Nematoda	66	22,679	20,000
Plathyhelminthes	42	18,040	25,000
Annelida	49	10,646	15,000
Echinodermata	25	8,760	7,000
Porifera	15	3,906	5,000
Other Metazoan phyla	43	58,593	20,395

**Table 2:** Results of database search in Zoological Record™ (1978–2006) for biogeographical regions. CSR = cryptic species reports.

**Region**	**N CSR**	**N articles**	**% species**
Neotropis	177	49,637	41.51
Afrotropis	148	30,705	22.18
Indomalayan	115	45,651	11.97
Nearctis	268	71,638	3.16
Palaearctis	447	213,501	5.38
Australasia + Oceania	184	38,711	15.78
Antarctis	15	5,813	0.02

## Authors' contributions

Both authors planned the study and drafted the manuscript. MP retrieved and analysed the data.
